# Lectures on the Theory and Practice of Physic, Delivered in the College of Physicians and Surgeons of the University of the State of New York

**Published:** 1838-12-05

**Authors:** 


### BIBLIOGRAPHICAL NOTICE.

*Lectures on the Theory and Practice of Physic, delivered in the College of Physicians and Surgeons of the University of the State of New York, by the late* David Hosack, M.D., LL.D., F.R.S., Professor of the Theory and Practice, &c., and of Clinical Medicine in that Institution. *With an Introductory Letter, by* Nathaniel Chapman, M.D., Professor of the Theory and Practice of Medicine in the University of Pennsylvania, &c. *Edited by his Friend and former Pupil,* Henry W. Ducachet, D.D., Rector of St. Stephen's Church, Philadelphia. Philadelphia: Herman Hooker, Chesnut Street. 1838.

Dr. Hosack's Lectures contain a lively and faithful description of the symptoms of fevers, chiefly derived from his own observations. The part of the course which is devoted to fevers, occupies a large portion of the volume; the rest is taken up with the phlegmasiæ, and some diseases which are allied to them. In this portion of the work we must expect some deficiencies. The diagnosis of diseases, by means of the local signs, has become much more perfect, at present, than during the life time of Dr. Hosack, and a series of lectures, which are based exclusively upon the general symptoms, now presents an incomplete and, as it were, an unfinished appearance.

It has often been stated that our knowledge of the general symptoms of disease has become much more accurate since the discoveries of Laenec, at least since their general adoption by physicians. This is abundantly shown by the present course of lectures. Dr. Hosack has not given so extended nor so precise an account of the general symptoms of pectoral disease, as are now to be found in most works specially devoted to the subject. An exclusive reliance on general symptoms does not render us more successful in discovering them; on the contrary, the general symptoms of disease are more completely studied since the attention of physicians has been directed to the local signs.

The transmissibility of diseases, by direct contagion, was a favourite doctrine of Dr. Hosack. We know not what may have been his doctrines in the earlier periods of his life, but it is certain that his lectures do not present his opinions as to contagion in a very exceptionable form. Indeed, with them we are disposed to coincide to a great extent, for we believe that certain diseases, when the patients are collected together in large numbers, do tend to reproduce themselves, as it were, in healthy individuals. We may call it a feeble degree of contagion which can only act when a large number of patients are thus concentrated together, or we may modify our phraseology still further, and term it infection; the truth remains, that but a few diseases, under unfavourable circumstances, particularly imperfect ventilation and the crowding of many individuals together, do tend to reproduce themselves. Amongst these diseases, are to be found typhus, scarlatina, and measles, and, very probably, typhoid fever, cholera, and, perhaps, in rare circumstances, yellow fever. Of course we do not now allude to those cases in which the contagious principle is very powerful, as variola and syphilis, but merely to a class of diseases which are often regarded as entirely insusceptible of direct transmission. The opponents of contagion in this country have emancipated themselves from many absurd prejudices; but while they have rejected numerous errors they have, perhaps, fallen into others of less importance than those which they have avoided, but still leading them away from the conclusions to which strict observation would have conducted them.

